# Datasets on the GRP of Russian regions, GRP sectoral composition and growth rates in 2013–2018

**DOI:** 10.1016/j.dib.2020.106551

**Published:** 2020-11-25

**Authors:** Gennady M. Fedorov, Tatyana Yu. Kuznetsova

**Affiliations:** Immanuel Kant Baltic Federal University, 236016, Kaliningrad, Russia

**Keywords:** Gross regional product (GRP), Economic development, GRP sectoral composition, GRP growth rates, Russian regions, Typology of regions

## Abstract

This paper presents a comparative analysis of the gross regional products (GRPs) of 85 Russian regions. Statistical methods were used to analyze datasets on regional GRP, its sectoral composition and growth rates. Many datasets have been computed for the first time, including those of gross value added per capita and per employee. The work reviews a range of evidence on the contribution of the production of goods and market and non-market services to regional GRPs. The production of goods was further analyzed by economic sectors for each region. The data used in computations were provided by the Russian Federal State Statistics Service (ROSSTAT). The authors built a typology of Russian regions based on GRP figures, GRP sectoral composition and growth rates. The data obtained make it possible to determine a set of indicators of the geographical distribution of Russia's economy and its growth rates. The findings are presented in the form of tables, maps and graphic materials (three tables, three charts and 11 cartographic maps), displaying quantitative measures of the economic performance of Russian regions .

## Specifications Table

**Table:** 

Subject	Geography, planning and development
Specific subject area	Economic geography
Type of data	3 Tables 13 Figures
How data were acquired	Data were acquired from the Russian Federal State Statistics Service (ROSSTAT) (Rosstat): EMISS database (https://fedstat.ru/)
Data format	Raw Analyzed Filtered
Parameters for data collection	The analysis is based on the statistical data on the gross regional product of Russian regions obtained from EMISS – a Rosstat electronic database. The data were subjected to initial statistical processing. When required, the arithmetic mean was used for computing GRP per capita. Chain indices were calculated to estimate GRP growth rates. A typology of Russian regions was developed using the SPSS statistical software. Groups of regions and the most important relative indicators characterizing GRPs of Russian regions were presented in the form of tables, charts and cartographic maps. The geographical distribution of Russian economic centers was illustrated by maps and cartographic diagrams. EMISS data on GRP per capita were used for calculating gross value added per capita and per employee for different economic sectors.
Description of data collection	The initial and computed data characterize the economic development of Russia and its regions. The collected data reflect GRP per capita figures, the percentage of goods, market and non-market services in the GRP sectoral composition, and GRP growth rates for different economic sectors. The data on population density, the percentage of the urban population, population income and wages were used for establishing correlations between GRP and other relevant socio-economic indicators.
Data source location	Russian Federation and its 85 regions: Belgorod region, Bryansk region, Vladimir region, Voronezh region, Ivanovo region, Kaluga region, Kostroma region, Kursk region, Lipetsk region, Moscow region, Orel region, Ryazan region, Smolensk region, Tambov region, Tver region, Tula region, Yaroslavl region, Moscow (Central Federal District); Republic of Karelia, Komi Republic, Arkhangelsk region without the Nenets autonomous region, Nenets autonomous region, Vologda region, Kaliningrad region, Leningrad region, Murmansk region, Novgorod region, Pskov region, St. Petersburg (Northwestern Federal District); Republic of Adygeya, Republic of Kalmykia, Republic of Crimea, Krasnodar region, Astrakhan region, Volgograd region, Rostov region, Sevastopol (Southern Federal District); Republic of Dagestan, Republic of Ingushetia, Kabardino-Balkarian Republic, Karachay-Cherkess Republic, Republic of North Ossetia – Alania, Chechen Republic, Stavropol region (North Caucasus Federal District); Republic of Dagestan, Republic of Ingushetia, Kabardino-Balkarian Republic, Karachay-Cherkess Republic, Republic of North Ossetia – Alania, Chechen Republic, Stavropol region (North Caucasus Federal District); Republic of Bashkortostan, Republic of Mari El, Republic of Mordovia, Republic of Tatarstan, Udmurt Republic, Chuvash Republic, Perm region, Kirov region, Nizhny Novgorod region, Orenburg region, Penza region, Samara region, Saratov region, Ulyanovsk region (Volga Federal District); Tyumen region (without the Khanty-Mansi autonomous region – Yugra and Yamal-Nenets autonomous regions); Khanty-Mansi autonomous region – Yugra, Yamal-Nenets autonomous region, Kurgan region, Sverdlovsk region, Chelyabinsk region (Ural Federal District);
Data source location	Republic of Altai, Republic of Tuva, Republic of Khakassia, Altai region, Krasnoyarsk region, Irkutsk region, Kemerovo region, Novosibirsk region, Omsk region, Tomsk region (Siberian Federal District). Republic of Buryatia, Trans-Baikal region, Republic of Sakha (Yakutia), Kamchatka region, Primorye region, Khabarovsk region, Amur region, Magadan region, Sakhalin region, Jewish autonomous region, Chukotka autonomous region (Far Eastern Federal District). Primary data sources are presented in Annex 1 -3. The list of the primary data sources is provided as a supplementary file (Annex 4)
Data accessibility	Data is uploaded to Mendeley Data http://dx.doi.org/10.17632/n36vrd8zrp.1

## Value of the Data

• This dataset provides a comprehensive picture of the geographical distribution of Russia's economy and allows researchers to conduct their own custom analysis of Russia's economy on regional level.
• A wider research community can benefit from the dataset that is larger and has more material classes describing the economic development of Russian regions.
• The data allow researchers to perform multiple statistical analyses by introducing other independent variables.
• The data can be used to perform a comparative analysis of the economic development of Russian regions and regions of other countries.
• Although the data were obtained from open sources they are little known to specialists.

## 1 Data Description

An important measure of regional performance, regional GDP is traditionally used for a comprehensive analysis of regional economies across the globe. In this work, we performed a comparative analysis of GRP output, GRP sectoral composition and growth rates of 85 Russian regions. These indicators have been extensively analyzed in economic literature. Many authors have built their typologies of regions based on GRP per capita [1], [2], [3], [4] whereas much less attention has been paid to the sectoral structure of GRP. There is a vast body of literature on the sectoral composition of GRP. The data for gross value added per capita and per employee within individual economic sectors tend, however, to be insufficiently researched [5], [6], [7], [8], [9].

This work presents a comprehensive large-scale assessment of the gross regional products of 85 Russian regions. The computations characterize the level of economic development of different parts of the country: GRP measures the value of goods and services produced in a region; GRP per capita reflects the economic output of a region and the standard of living; the ratio of different economic sectors defines the sectoral composition of regional economies. Special attention was paid to the analysis of datasets for the production of goods, market and non-market services.

The sectoral composition of GRP was analyzed using two datasets – on GRP per capita and GRP growth rates. This makes it possible to evaluate the economic performance of individual sectors of the regional economy and to measure labor productivity and economic growth rates.

Cartographic representation of GRP helps reveal similarities and disparities in regional economic development.

Types and subtypes of regions were identified based on the following coupled indicators:

1) GRP per capita and employee – gross value added per sector (per type of economic activity);
2) GRP sectoral composition of consolidated groups of economic activity (production of goods, market and non-market services) – the production of goods per economic sector
3) GRP growth rates - GRP per capita.

The initial and computed statistical data are presented in three tables, two diagrams and 10 cartographic maps for all 85 regions of the Russian Federation. They illustrate GRP figures and sectoral composition in 2018 and the growth rates of GRP per capita during two periods – 2004–2018 and 2013–2018.

The economic differentiation of Russian regions is analyzed according to 30 indicators.

The aggregated GRP and social indicators datasets on Russian regions are available in spreadsheets (Annex 1).

The aggregated GRP per economic sectors in 2018 datasets on Russian regions are available in separate spreadsheets (Annex 2).

Annex 3 contains tables presenting groups of Russian regions formed according to the chosen indicators (see Fig. 3, Fig. 4,Fig. 6, Fig. 7, Fig. 8, Fig. 9, Fig. 10, Fig. 11, Fig. 12, Fig. 13, Fig. 14).

**Fig. 1:**
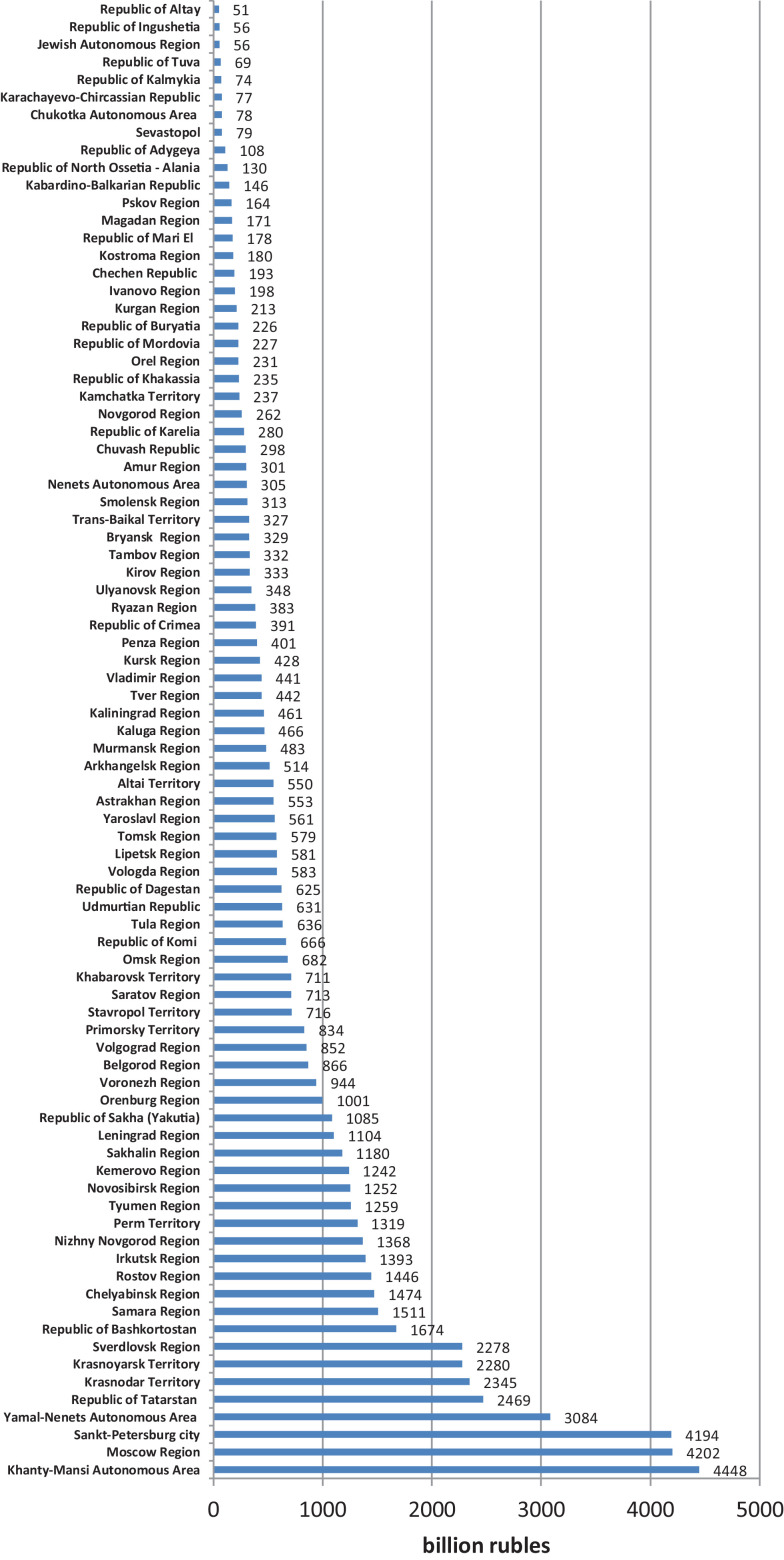
GRP ranking of Russian regions, billion rubles, 2018.

Fig. 1 presents a region-by-region comparison of GRPs.

**Fig. 2:**
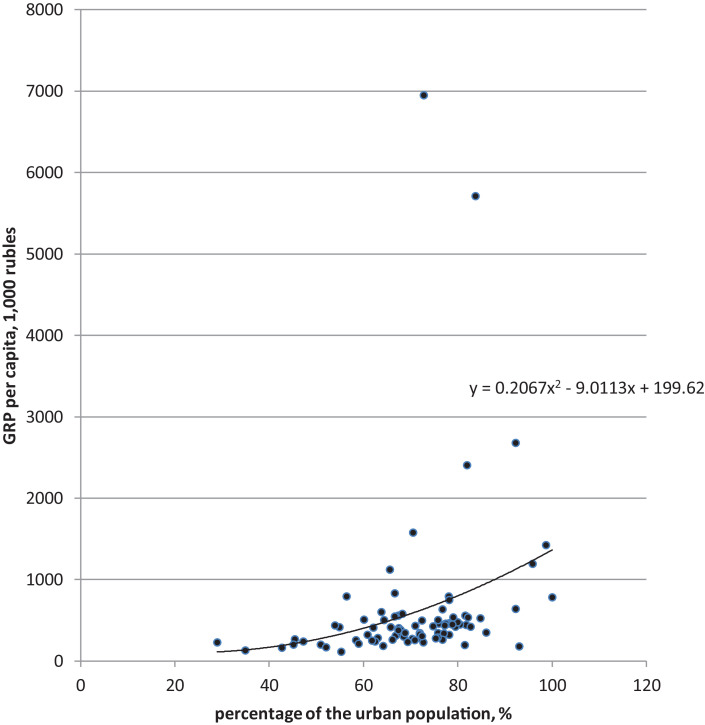
Grouping of Russian regions according to the percentage of the urban population and GRP per capita, 2018.

**Fig. 3:**
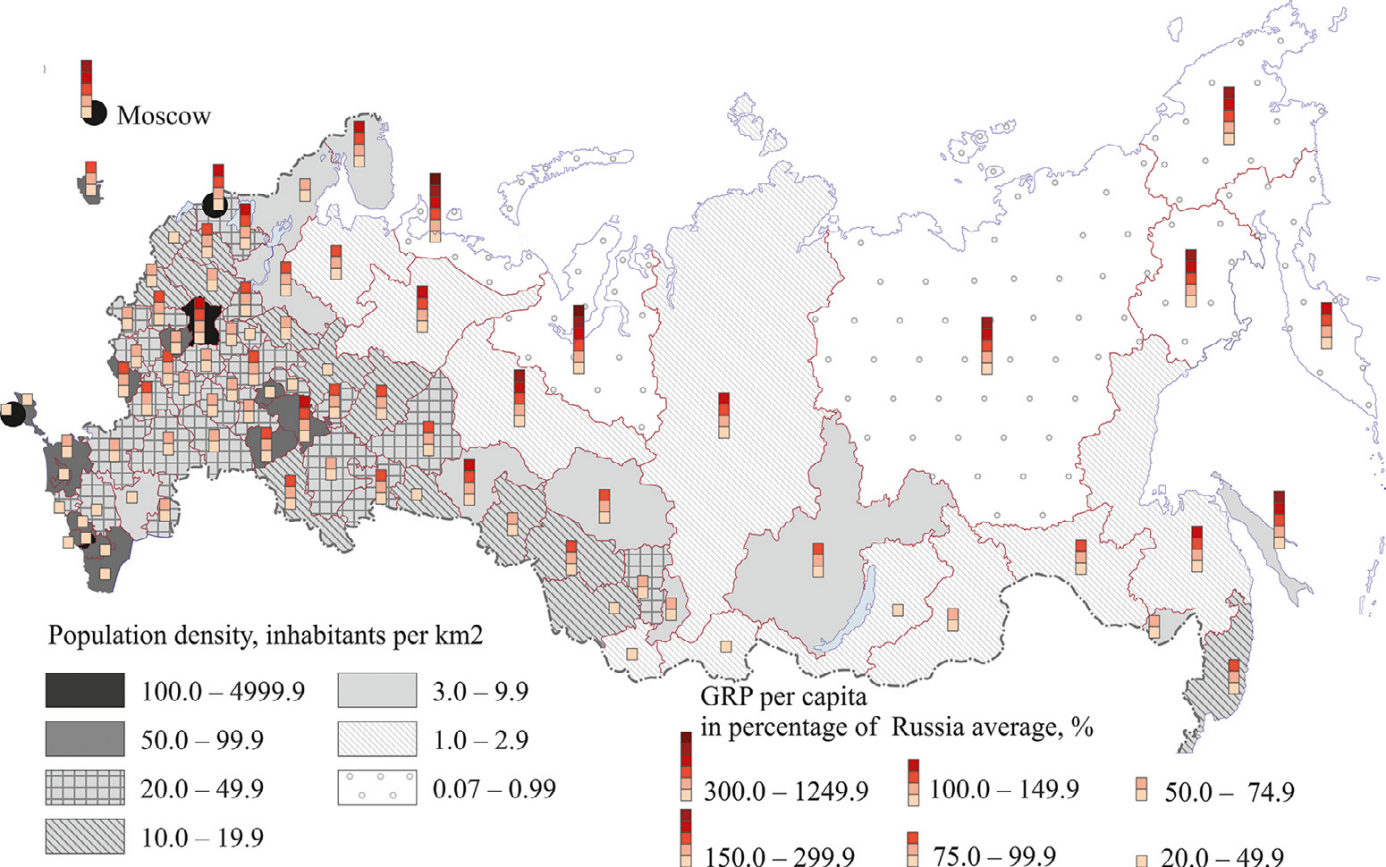
Russian regions: population density and GRP per capita.

Fig. 2, Fig. 3 show the correlation between population numbers and GRP per capita.

**Fig. 4:**
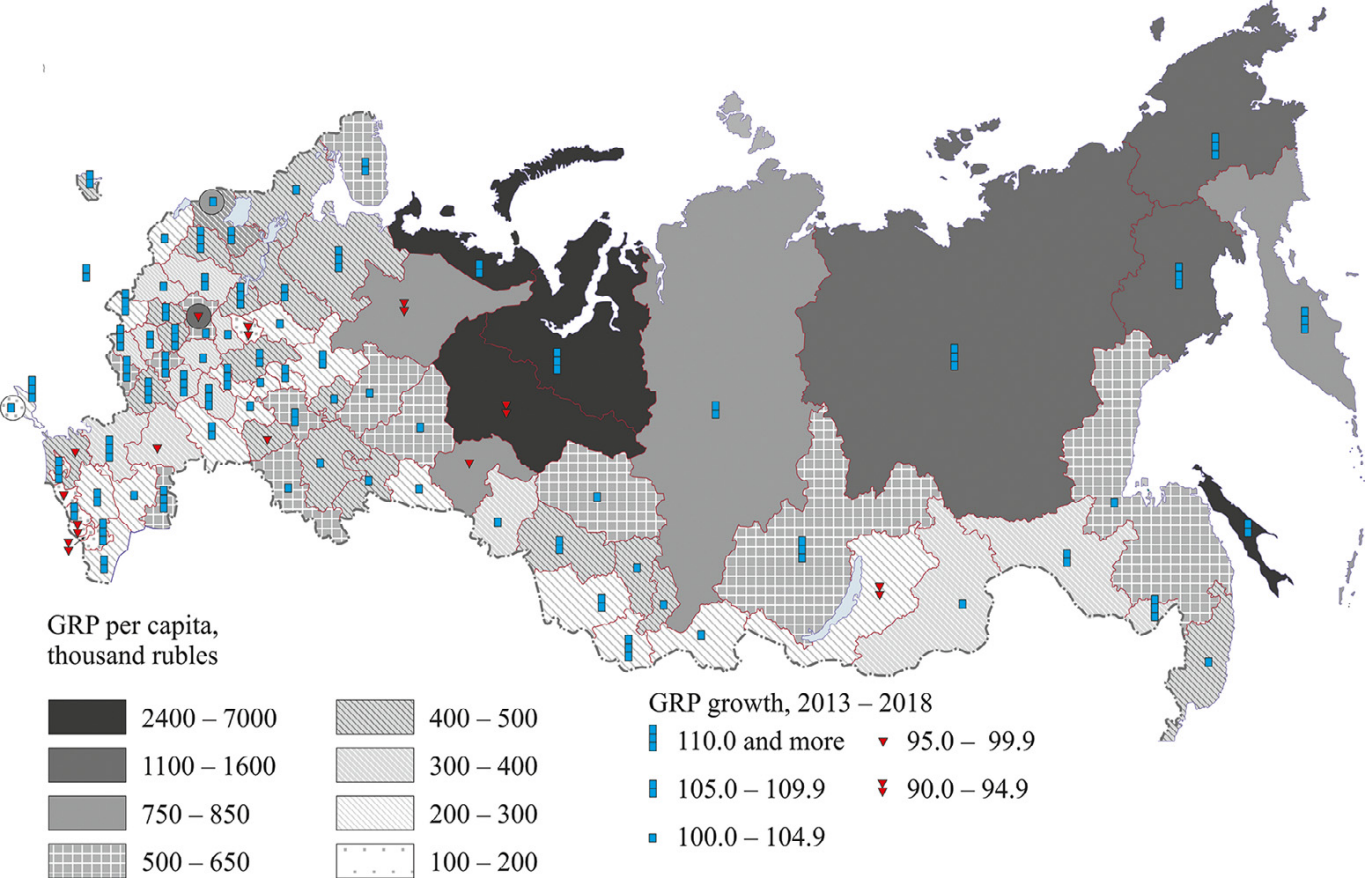
GRP per capita and GRP growth rates.

Fig. 4 demonstrates GRP per capita and GRP growth rates.

Fig 5, Fig. 6, Fig. 7, Fig. 8, Fig. 9, Fig. 10, Fig. 11, Fig. 12, Fig. 13, Fig. 14 represent GRP sectoral composition.

**Fig 5:**
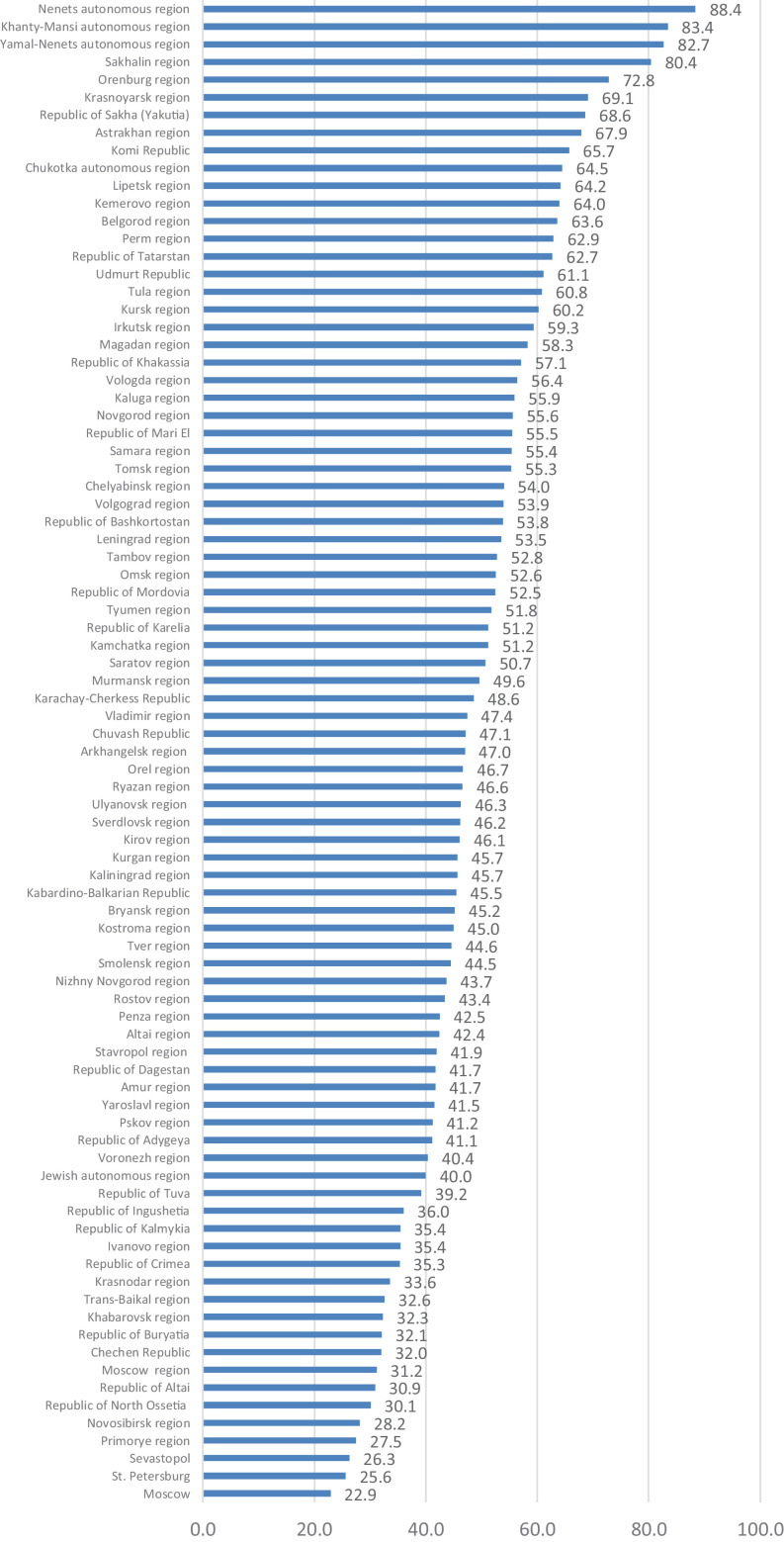
The share of the production of goods in GRP sectoral composition, %, 2018.

Table 1 displays the ranking of Russian regions according to GRP per capita and employee.

**Table 1:** Group ranking of Russian regions according to GRP per capita and per employee, thousand rubles (2018)

Regions	GRP per capita	GRP per employee
1. Nenets autonomous region, Khanty-Mansi autonomous region, Yugra, Yamal-Nenets autonomous region, Sakhalin region	2000 – 6999	4000 – 9999
2. Moscow city, Republic of Sakha (Yakutia), Magadan region, Chukotka autonomous region	1000 – 1999	1500 – 4999
3. Komi Republic, Krasnoyarsk region, Kamchatka, Tyumen region	500 – 999	500 – 2199
4. St. Petersburg, Republic of Tatarstan, Perm region, Belgorod region, Moscow region, Leningrad region, Murmansk region, Astrakhan region, Sverdlovsk region, Irkutsk region, Tomsk region	500 – 999	1100 – 1499
5. Republic of Karelia, Republic of Bashkortostan, Udmurt Republic, Republic of Khakassia, Krasnodar region, Khabarovsk region; Kaluga region, Lipetsk region, Yaroslavl region, Arkhangelsk region, Vologda region, Kaliningrad region, Novgorod region, Orenburg region, Samara region, Kemerovo region, Novosibirsk region	400 – 549	90 – 1099
6. Trans-Baikal region, Primorye region, Vladimir region, Voronezh region, Kursk region, Orel region, Ryazan region, Smolensk region, Tambov region, Tver region, Tula region, Tula region, Rostov region, Nizhny Novgorod region, Penza region, Chelyabinsk region, Omsk region, Amur region, Jewish autonomous region	300 – 449	500 – 899
7. Republic of Adygeya, Republic of Kalmykia, Republic of Daghestan, Republic of Mari El, Republic of Mordovia, Chuvash Republic, Republic of Altay, Republic of Tuva, Republic of Buryatia, Stavropol region, Altay region, Bryansk region, Kostroma region, Pskov region, Kirov region, Saratov region, Ulyanovsk region, Kurgan region	200 – 299	500 – 899
8. Sevastopol, Republic of Crimea, Republic of Ingushetia, Kabardino-Balkarian Republic and Karachay-Cherkess Republics, Republic of North Ossetia - Alania, Chechen Republic, Ivanovo region	100 – 205	300 – 499

Table 2 shows groups of Russian regions categorized according to GRP, GRP sectoral composition and per capita growth rates.

**Table 2:** Types of economic activities. OKVED 2 (2018)

Production of goods
A. Agriculture, forestry, hunting, fishing and fish farming
B. Extraction of mineral resources
C. Manufacturing industries
D. Electricity, gas and steam supply; air conditioning
E. Water supply, sanitation, waste collection and disposal, pollution elimination
F. Construction
Market services
G. Wholesale and retail trade; repair of motor vehicles and motorcycles
H. Transportation and storage
I. Hotels and catering
J. Information and communication
K. Financial and insurance
L. Real estate operations
Production of non-market services
M. Professional, research and technical activity
N. Administration, management and related auxiliary services
O. Public administration and military security, social security
P. Education
Q. Health and social services
R. Culture, sport, leisure and entertainment
S. Other services

Regions of the Russian Federation differ greatly in size, natural resources, the number and replacement rate of the population, GRP figures and other socio-economic indicators. For instance, Sevastopol, a city of federal importance, (territory – 0.9 thousand sq. km) is 3.4 thousand times smaller than the Republic of Sakha (Yakutia) (3083.5 thousand sq. km). The population of the Nenets autonomous region (44,000 people) is 288 times smaller than that of Moscow (12,692,000 people). In 2018, the GRP of the Republic of Altai amounted to 51 billion rubles, whereas that of Moscow to 17 882 billion rubles, which is 351 times as much. In 2018, the highest average annual growth rate of GRP per capita as compared to 2013 was 6.5% (in the Yamal-Nenets autonomous region), and the largest decrease was -2.0% in the Republic of North Ossetia-Alania.

Fig. 1 and Annex 1 shows the GRP ranking of Russian regions

The GRP of Moscow is four times that of the Khanty-Mansi autonomous region, which is the second-largest oil-producing region in Russia. The Moscow region (forming a single agglomeration with the city of Moscow) and St. Petersburg, which is often referred to as the second or cultural capital of the Russian Federation (in 1712 – 1918, St. Petersburg was the capital of Russia), have almost reached the GRP of Moscow.

The data in Table 1 reflect disparities between Russian regions in GRP per capita and per employee (see also Annex 1). These indicators are closely related (the computed linear correlation coefficient between them is 0.99). Their highest values are characteristic of the main oil and gas producing regions included in Group 1. The table below shows a group ranking of Russian regions according to GRP per capita and employee. Regions in Group 2 have lower per capita and employee figures than those in Group 1, but they are still significantly higher than those of Moscow and sparsely populated northern industrial regions – the Republic of Yakutia (Sakha), the Magadan region and the Chukotka autonomous region. Group 8 includes regions having the lowest per capita and employee values: five republics of the North Caucasus, the Ivanovo region (in the Soviet time it was an industrially developed textile region), located in the Non-Chernozem zone of Russia, and the Republic of Crimea and Sevastopol, which became part of Russia in 2014.

We established correlations between GRP per capita of Russian regions and some other socio-economic indicators. The coefficient of linear correlation of GRP per capita with urban population figures and population density is extremely small – 0.25 and 0.08 respectively.

Although many highly urbanized regions of Central Russia and the Volga region have rather low GRP per capita, the Moscow agglomeration boasting a very high level of urbanization is the social and economic nucleus of the country. The St. Petersburg agglomeration also plays an important role.

Fig. 2 shows the grouping of Russian regions according to the share of the urban population and GRP per capita (see also Annex 1). An increase in the percentage of the urban population leads to a rise in GRP per capita. The more urbanized the region, the higher GRP per capita. There is a certain correlation between the two considered indicators (with degree 2: y =  0.2067 × 2 - 9.0113x + 199.62). There are considerable disparities among regions both in terms of the level of urbanization and GRP per capita.

Fig. 3 shows the difference between regions in population density and GRP per capita (see also Table 1 (Annex 3)). The south of the European part of the country (to the south of the latitude of St. Petersburg) and the southern regions of Western Siberia are the most densely populated. Yet, GRP per capita is higher in northern and eastern regions (except those bordering Mongolia). This part of the country has low population numbers and abundant mineral and forest resources. Regions located on the Pacific coast of Russia boast considerable marine fish resources. This results in high GRP per capita. More industrialized and more populated regions have higher than average GRP per capita even though they have no mining or logging industries. Many border regions – the republics of North Caucasus, Crimea and the Pskov region, located on the periphery, are lagging. The Mari El and Chuvash Republics in the Middle Volga region, Stavropol region, Ivanovo and Kirov regions also have low GRP per capita.

Fig. 4 presents variations in GRP per capita and its growth rates over five years (2013–2018)

The correlation between these two indicators is rather low: the linear correlation coefficient is 0.14. As Fig. 2 shows, regions belonging to the same group, i.e. having almost the same GRP per capita, may have completely different GRP growth rates. For example, the Yamal-Nenets and Khanty-Mansi autonomous regions, which have the highest GRP per capita in their federal district, differ considerably in GRP growth rates: the Yamal-Nenets region has high GRP growth rates, whereas the Khanty-Mansi region has decreasing GRP per capita. Regions having particularly low per capita figures demonstrate the same tendency: a massive decline in GRP in the Republics of North Ossetia-Alania and Ingushetia and the Ivanovo region versus a more modest decline in the Republic of Karachay-Cherkess Republic; a small increase in Sevastopol versus a sharper decline in the Kabardino-Balkarian Republic and the rapid decline observed in the Chechen Republic (Fig. 4; Annex 1).

Of all federal districts of Russia, only the regions of the Far East saw an increase in GRP per capita.

In the Siberian Federal District, GRP per capita decreased in the analyzed period. The decrease was particularly dramatic in the Republic of Buryatia (7%), which had already had very low per capita figures. In the Ural Federal District, there are two territories, the Tyumen Oblast and the Khanty-Mansi autonomous region, which belong to regions with high GRP per capita.

Two regions in the Southern Federal District and three in the North Caucasus (where GRP per capita is traditionally below the country's average) also have declining GRP figures. At least one region saw a decline in GRP per capita in the Central, Northwestern and Volga Federal Districts.

Further research requires analysis of the GRP sectoral composition. Table 2 shows types of economic activity according to the All-Russia Register of Economic Activities as of 2018 (OKVED 2), approved in 2014 with subsequent changes. We divided all economic activities into three large groups: production of goods, market and non-market services. The terms “market” and “non-market” services are conventional, although both refer to the market. Non-market services are services provided for a fee and focused not only on cost recovery but also on revenue generation. Non-market services are mainly provided by government or municipal authorities.

Table 2 shows the GRP composition consisting of two groups (types) of economic activity – the production of goods and the production of services. The production of goods includes six types of economic activity (A–F). Dataset GRP per economic sectors in 2018 on Russian regions are available in Annex 2. There is an obvious correlation between GRP per capita and the share of the production of goods in the GRP structural composition (the linear correlation coefficient is 0.57). To illustrate, Moscow and St. Petersburg, which are the most economically developed subjects of the Russian Federation, have the lowest share of goods in GRP, 22.9% and 25.6% respectively.

The main Russian oil and gas regions have the largest share of the production of goods in their GRP structure: the Nenets (88.4%), Khanty-Mansi (83.4%) and Yamal-Nenets autonomous regions (82.7%) and Sakhalin (80.4%) (See Fig. 5; Table 10 Annex 3). The share of the production of goods in the Nenets autonomous region is almost four times that of the region having the lowest share (Moscow).

**Fig. 6:**
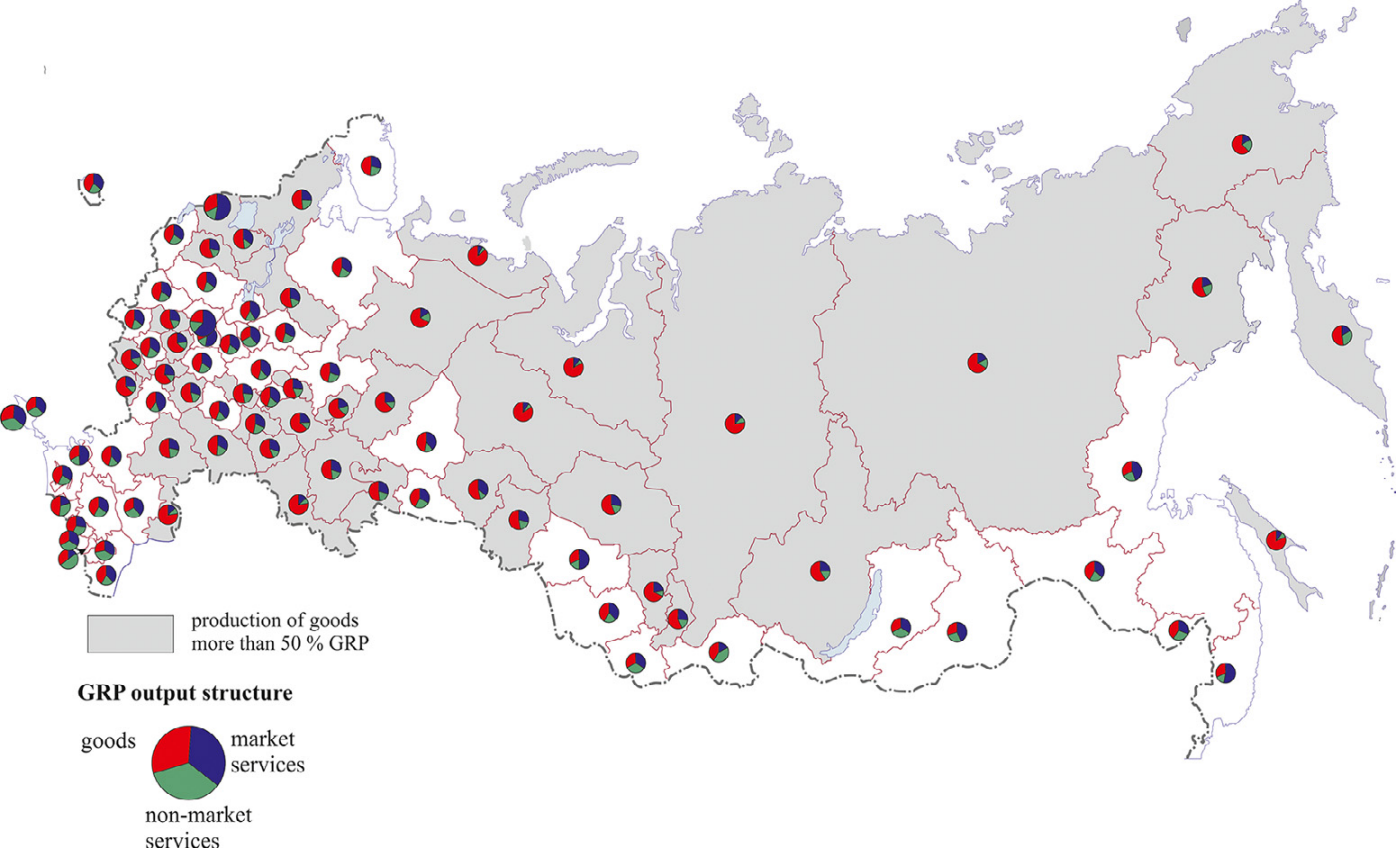
GRP composition, 2018.

Fig. 6 illustrates in more detail regional differences in the GRP sectoral composition. In most northern regions (except the Arkhangelsk and Murmansk regions) the share of the production of goods exceeds 50% (see also Table 9 Annex 3). This is also the case in almost the whole Volga region, the south of the Central Federal District, and the majority of regions of Russia's north-west. There are no such regions in the North Caucasus, in the south of Siberia or the Far East.

The analysis of the share of services shows that there are many regions where services dominate over the production of goods: Moscow (63%), St. Petersburg (54%) and the Moscow (53%), Primorye (52%) and Novosibirsk (51%) regions. The highest percentage of non-market services (33%–50%) is typical of Russia's national republics – Ingushetia (50%), Chechnya, North Ossetia-Alania, the Karachay-Cherkess Republic in the North Caucasus, Altai, Tyva, Buryatia in southern Siberia, as well as of cities of national importance (Sevastopol). This can be explained by a weaker development of the other two sectors – the production of goods and market services.

**Fig. 7:**
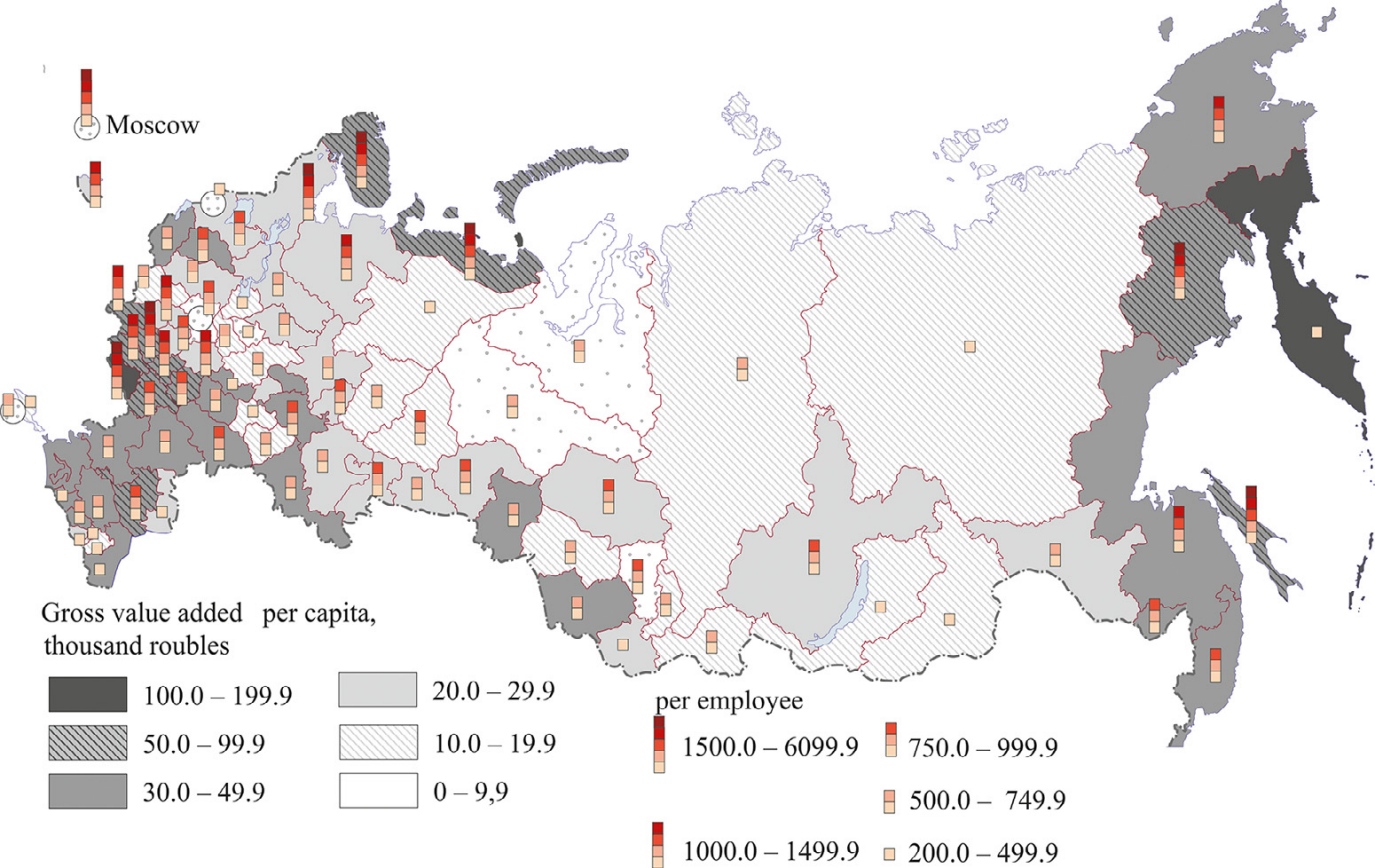
Gross value added per capita and per employee. Agriculture, forestry, hunting, fishing and fish farming (2018).

**Fig. 8:**
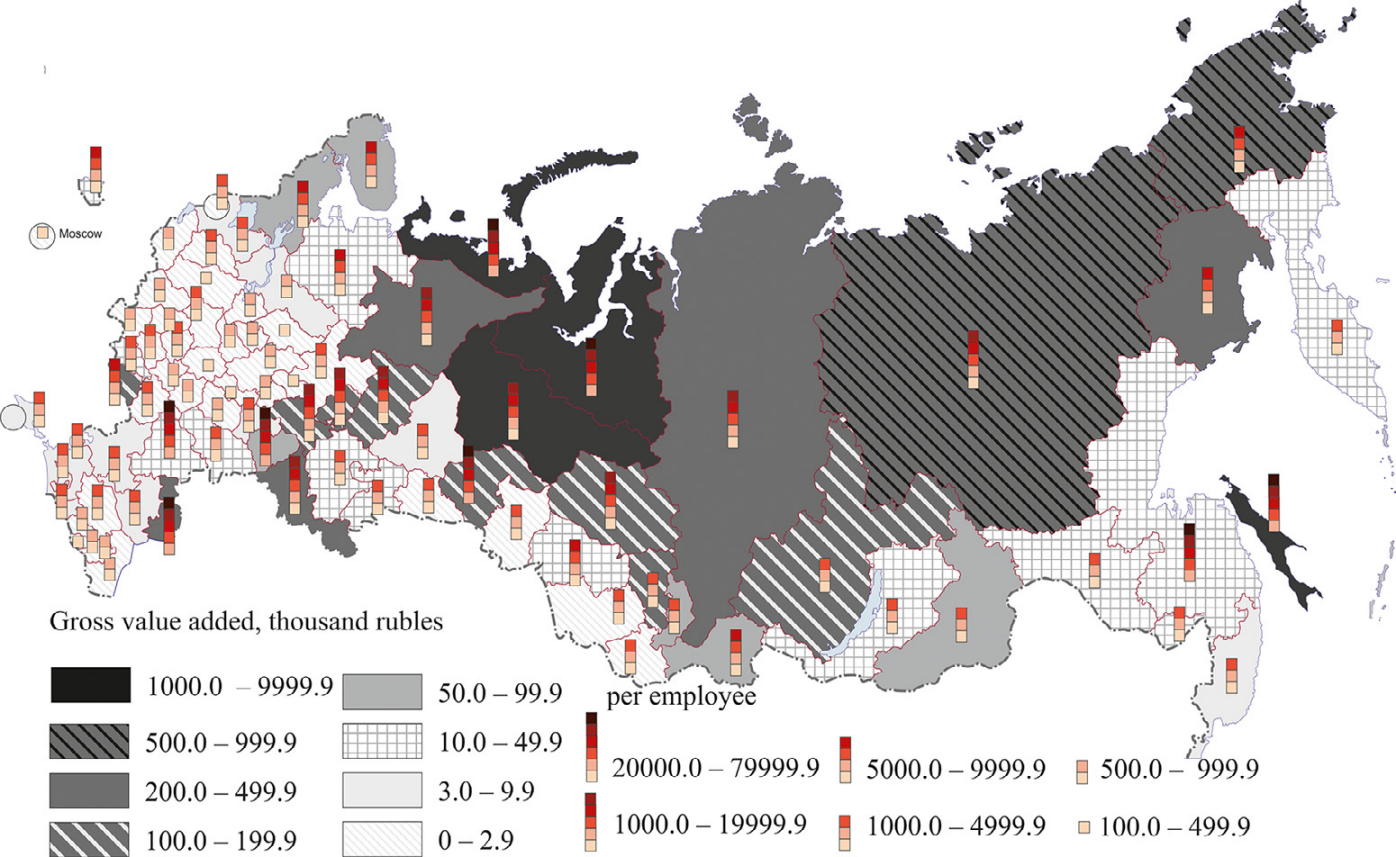
Gross value added per capita and per employee. Extraction of minerals, 2018.

**Fig. 9:**
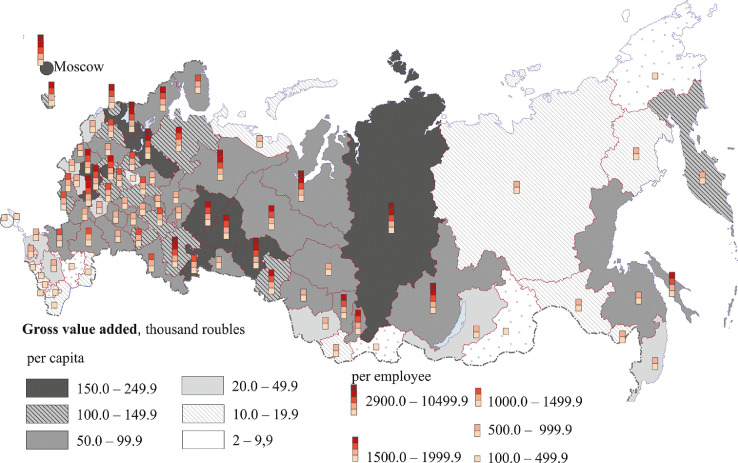
Gross value added per capita and per employee. Manufacturing, 2018.

**Fig. 10:**
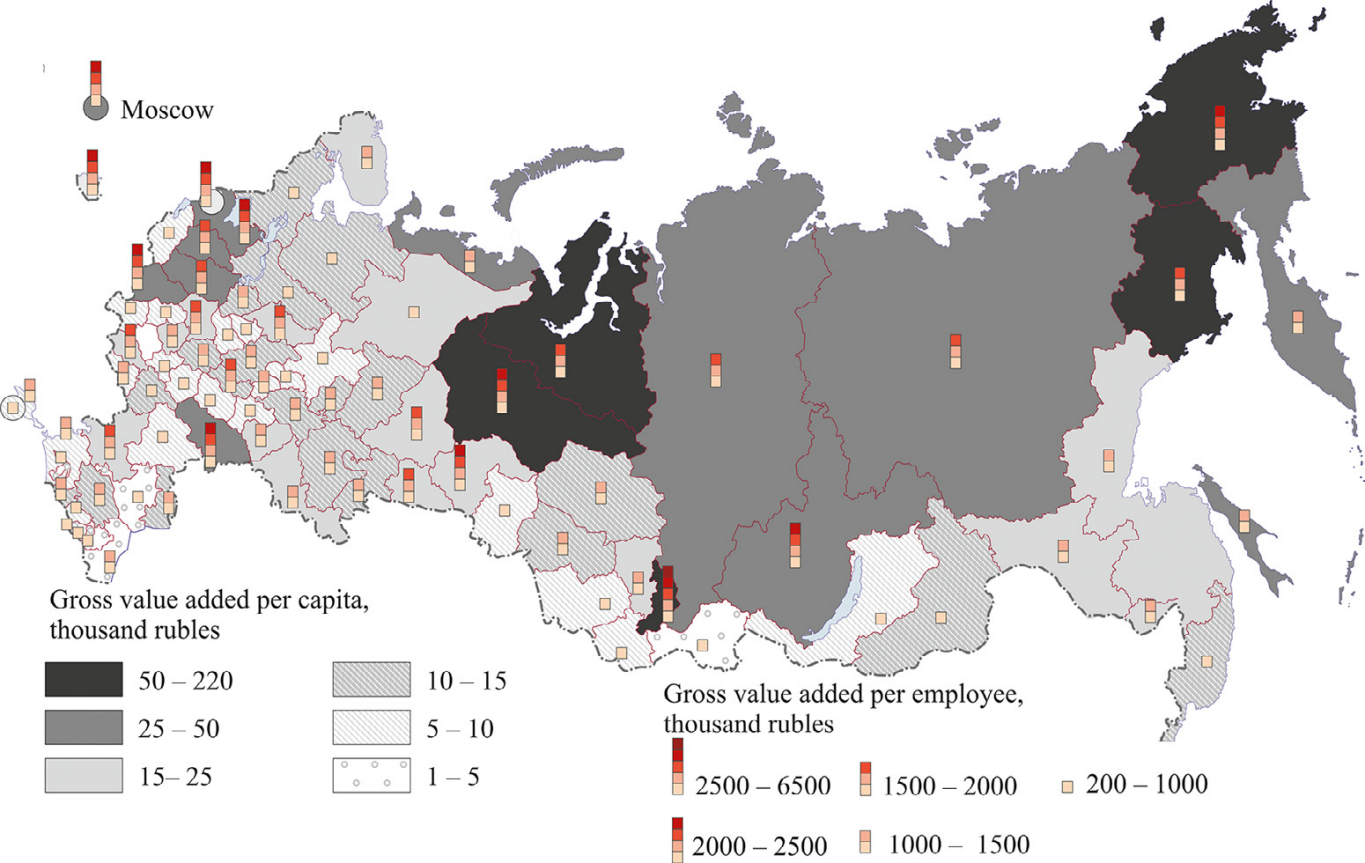
Gross value added per capita and per employee. Production of electric energy, gas, steam, air conditioning, 2018.

**Fig. 11:**
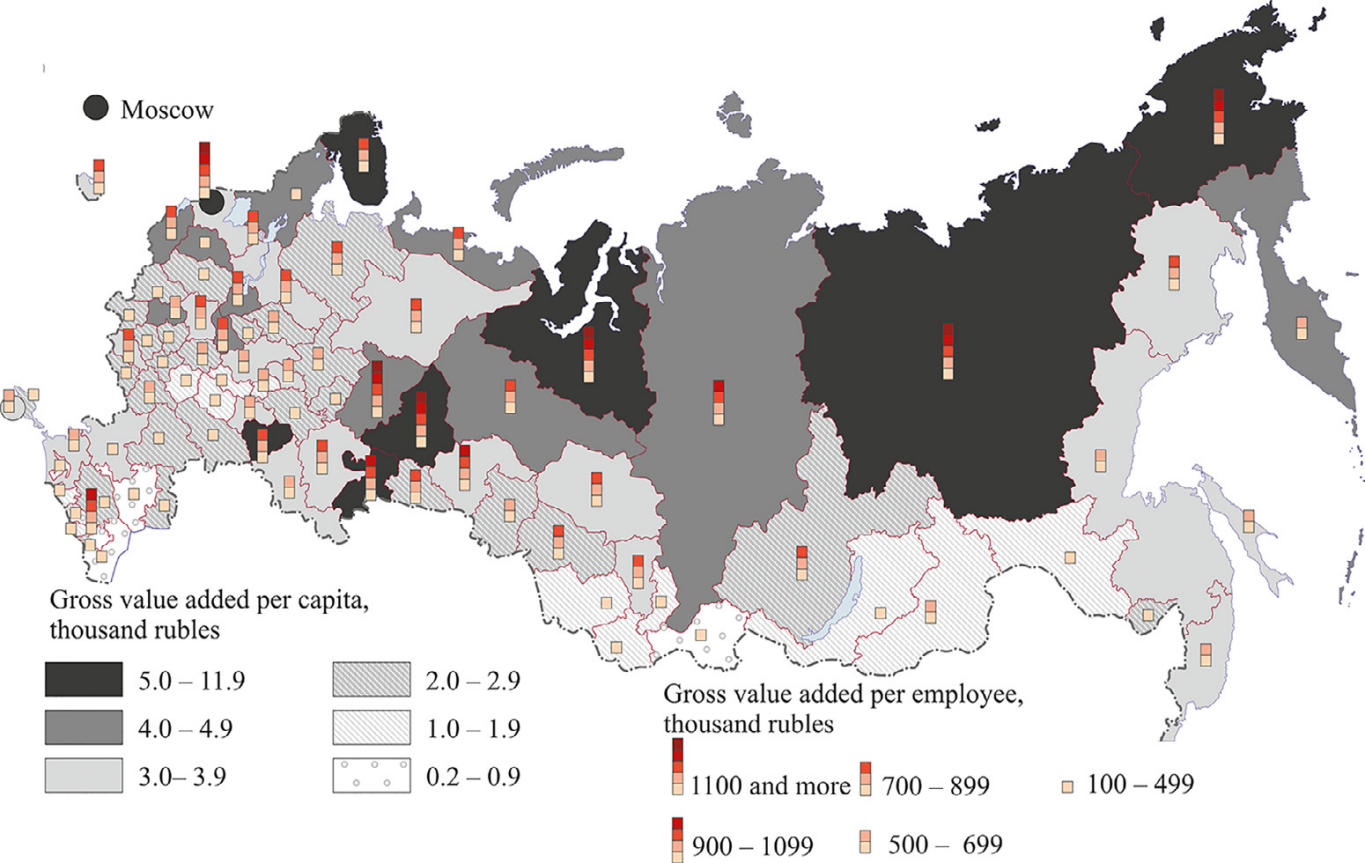
Gross value added per capita and per employee. Water supply, wastewater treatment, waste collection and disposal, pollution elimination, 2018.

**Fig. 12:**
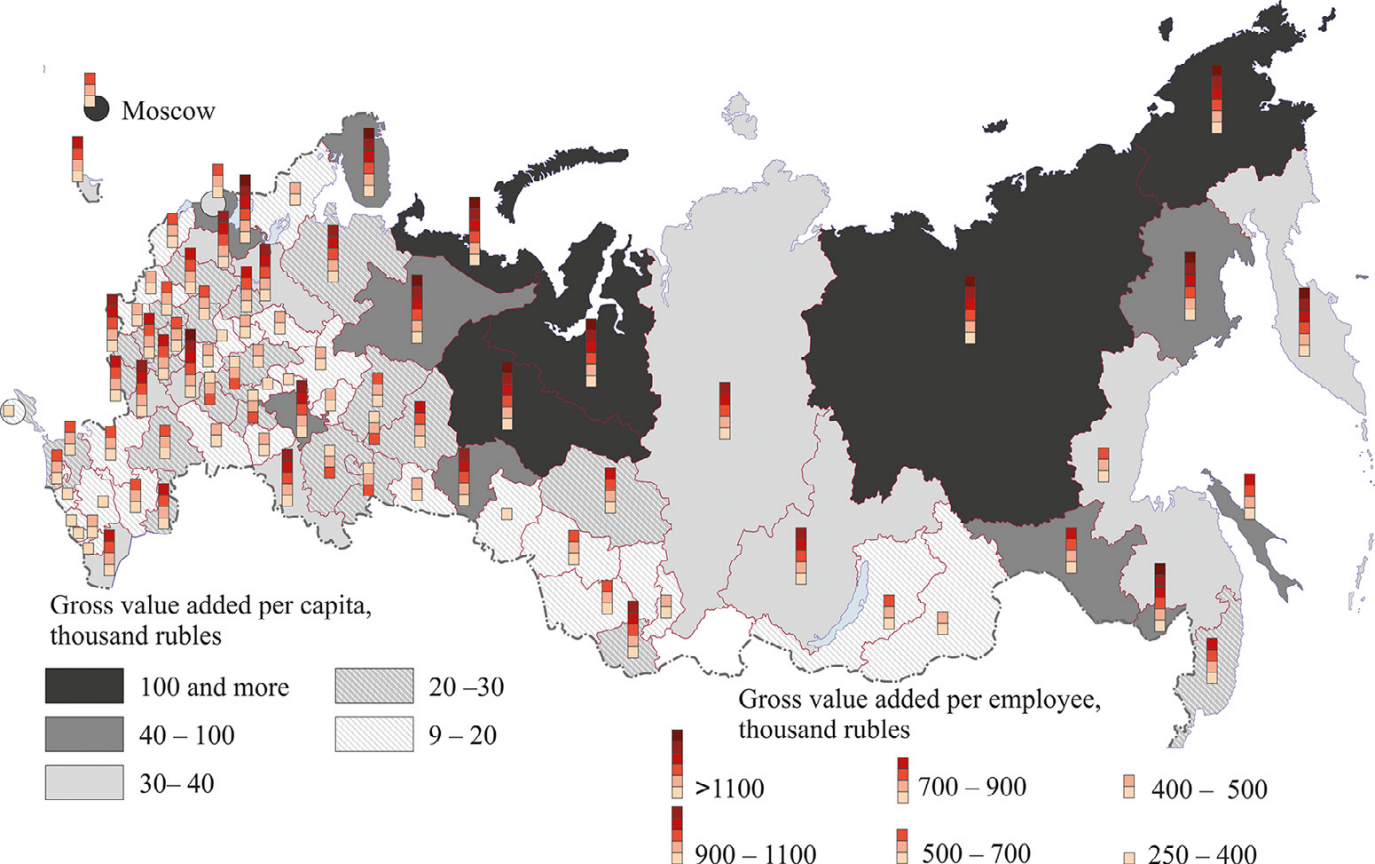
Gross value added per capita and per employee. Construction. 2018.

Fig. 7, Fig. 8, Fig. 9, Fig. 10, Fig. 11, Fig. 12 show GRP per capita and employee for industries related to the production of goods (see also Tables 2 -7 Annex 3).

Fig. 7 presents data for agriculture, forestry, hunting, fishing and fish farming.

The Belgorod region, located in the Chernozem zone of Russia, has fertile soils and is well developed industrially. The region has the highest value added both per capita and per employee. Other regions of the Chernozem zone – Orlov, Kursk, Lipetsk, Voronezh, Tambov and Bryansk (the latter belongs to the Non-Chernozem zone and has intensive farming) are catching up.

Regions of the North Caucasus (especially the Krasnodar region), Volga regions (the Republics of Tatarstan and Mari El, the Saratov region) and the Novgorod region are characterized by a relatively high GRP per capita. High per capita figures of some coastal regions (Murmansk, Magadan, Sakhalin, and Kamchatka) can be attributed to the development of fishing and fish farming. The Primorye region also has highly developed forestry and logging. Many northern and eastern regions having relatively high gross value added figures (the Republic of Karelia and the Arkhangelsk, Vologda, Irkutsk, and Khabarovsk regions) employ more people in forestry and logging than in agriculture. Territories with the least favorable natural conditions have poorly developed agriculture. These are the Arctic coastal regions of Siberia and the Far East, regions bordering Mongolia and the regions of the Northern Urals. Crimea and some regions of the Middle Volga region, Central and North-West of the European part of Russia have low value added figures.

Fig. 8 shows the extraction of minerals. Russia's mining regions are mainly located in the north and east of the country.

The highest value added per capita (over 1 million rubles) and per employee (over 10 million rubles) was registered in the main oil and gas regions – Sakhalin region and the Khanty-Mansi and Yamal-Nenets autonomous regions. The Republic of Sakha (Yakutia) and the Chukotka autonomous region have high value added per capita ranging from 500 thousand to 1 million rubles, and per employee – from 5 million to 20 million rubles. All these regions, except the Nenets autonomous region, are located in Siberia and the Far East. In the Krasnoyarsk (Eastern Siberia) and Magadan regions (Far East), the Orenburg region (Southern Urals), the Komi Republic (North-West) and the Astrakhan region (Lower Volga region - Southern Federal District), value added per capita and per employee exceeds 200,000 rubles and 5 million rubles respectively. Central Russia (except the Belgorod region), the southern part of Russia's north-west, the east of the Volga region, North Caucasus and some southern regions of Western Siberia have the lowest per capita and employee figures.

The manufacturing industry of Russia employs 14% of the working population. A higher percentage is observed only in trade and the repair and maintenance of motor vehicles and motorcycles (19%). The most developed manufacturing regions having high gross value added per capita and per employee are located in Central Russia and in the south of Russia's north-west, the Middle Volga region, in the Urals, as well as in the eastern regions of the country – primarily in the Krasnoyarsk region and the Tyumen region (see Fig. 9). Most eastern and northern regions, the south of Russia (especially the North Caucasus and the Crimea), some regions of Central Russia (Ivanovo, Bryansk, Tambov) and North-West (Pskov) have poorly developed manufacturing industries, although in the Soviet period these regions had large machine-building and consumer goods manufacturing enterprises.

Northern and eastern regions take the lead in the production of electric energy, gas, steam and air conditioning. This can be attributed to severe climatic conditions, which require considerable investment in the production of electric energy and heat for industrial and household needs (Fig. 10). There are two more groups of regions having high energy generation and consumption figures: 1) Moscow and the Moscow region, St. Petersburg and the Leningrad region and 2) regions having large power plants producing electric energy not only to cover their own needs but also to export it to other parts of the country (the Krasnoyarsk, Irkutsk, Smolensk, Saratov, and Tver regions).

Water supply, wastewater treatment, waste collection and disposal and pollution elimination are most developed in the northern regions of Russia having cold climate – in the Republic of Sakha (Yakutia), the Murmansk region, the Yamal-Nenets and Chukotka autonomous regions) and in the most economically developed and highly urbanized regions (Moscow, St. Petersburg and the Sverdlovsk, Chelyabinsk and Samara regions). These industries are the least developed in the republics of North Caucasus, southern Siberia and the Far East (Fig. 11).

In construction and the production of goods, value added per capita differs from region to region (Fig. 12). There is also a considerable difference between the regions in gross value added per employee, even within the same group of regions having similar gross value added per capita. This can be explained by the difference in the volume of housing construction, administrative and more investment-intensive industrial construction, as well as by different climatic and natural conditions of the regions. Russia's northern regions, especially the Republic of Yakutia (Sakha), the Nenets, Khanty-Mansi, Yamal-Nenets and Chukotka autonomous regions boast the highest per capita and employee figures. The Komi Republic, the Amur, Magadan and Sakhalin regions and the Jewish autonomous region report only slightly lower figures.

Moscow, the Republic of Tatarstan, and the Leningrad region have the highest indicators among industrially developed regions.

**Fig. 13:**
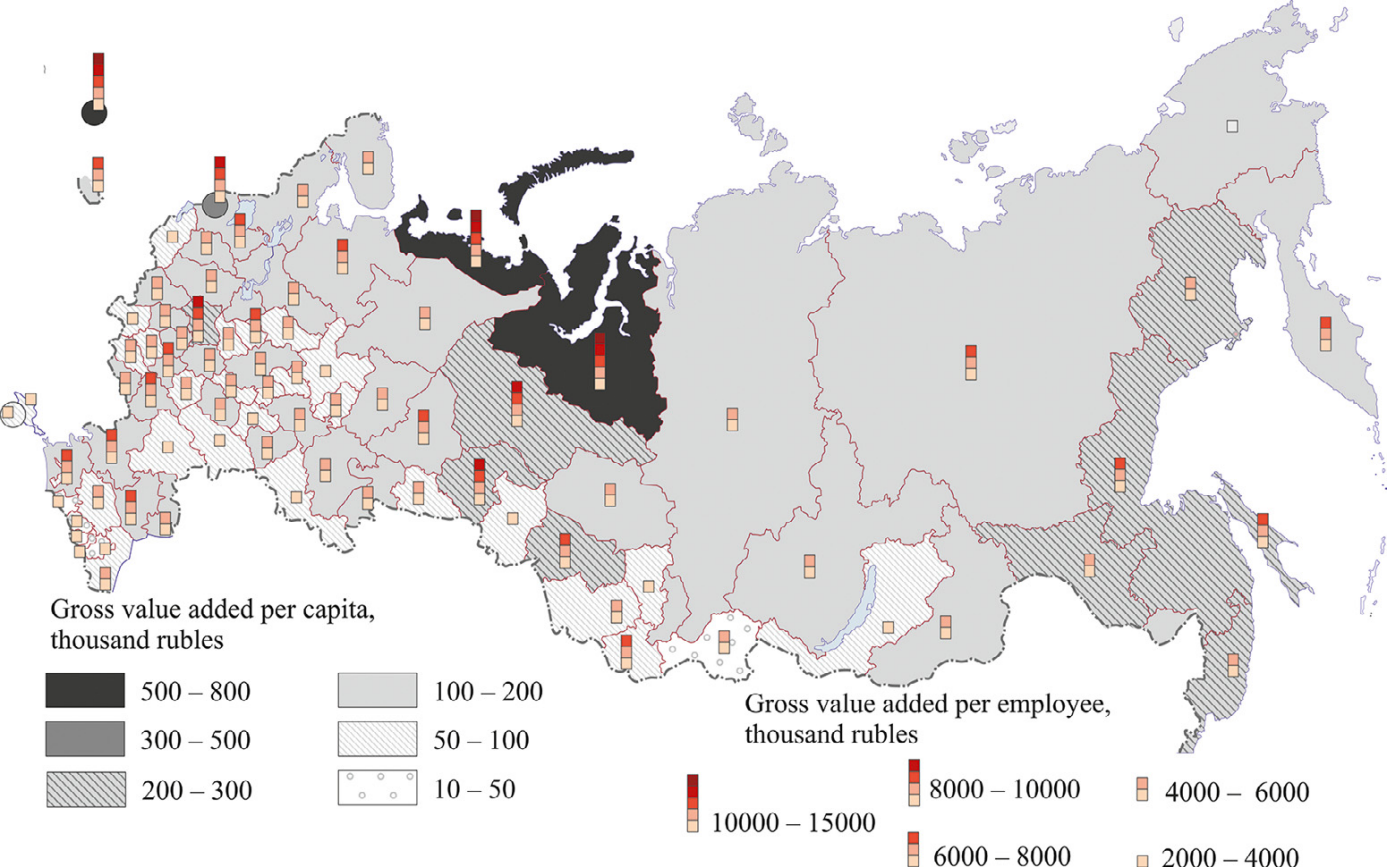
Gross value added per capita and per employee. Market services, 2018.

Among market services, the share of trade and repair of motor vehicles and motorcycles in the GRP sectoral composition is 44%; that of transportation and storage, 17%. The development of these sectors largely determines the value added per capita and per employee, which is the highest (as is the case for the production of goods) in the two capital cities, Moscow and St. Petersburg, and in the main oil and gas regions, the Nenets, Khanty-Mansi and Yamal-Nenets autonomous regions (see Fig. 13, Table 8 Annex 3). The Tyumen region, which includes the Khanty-Mansi and Yamal-Nenets autonomous regions, is also part of this group of regions and is home to many companies specializing in market services.

**Fig. 14:**
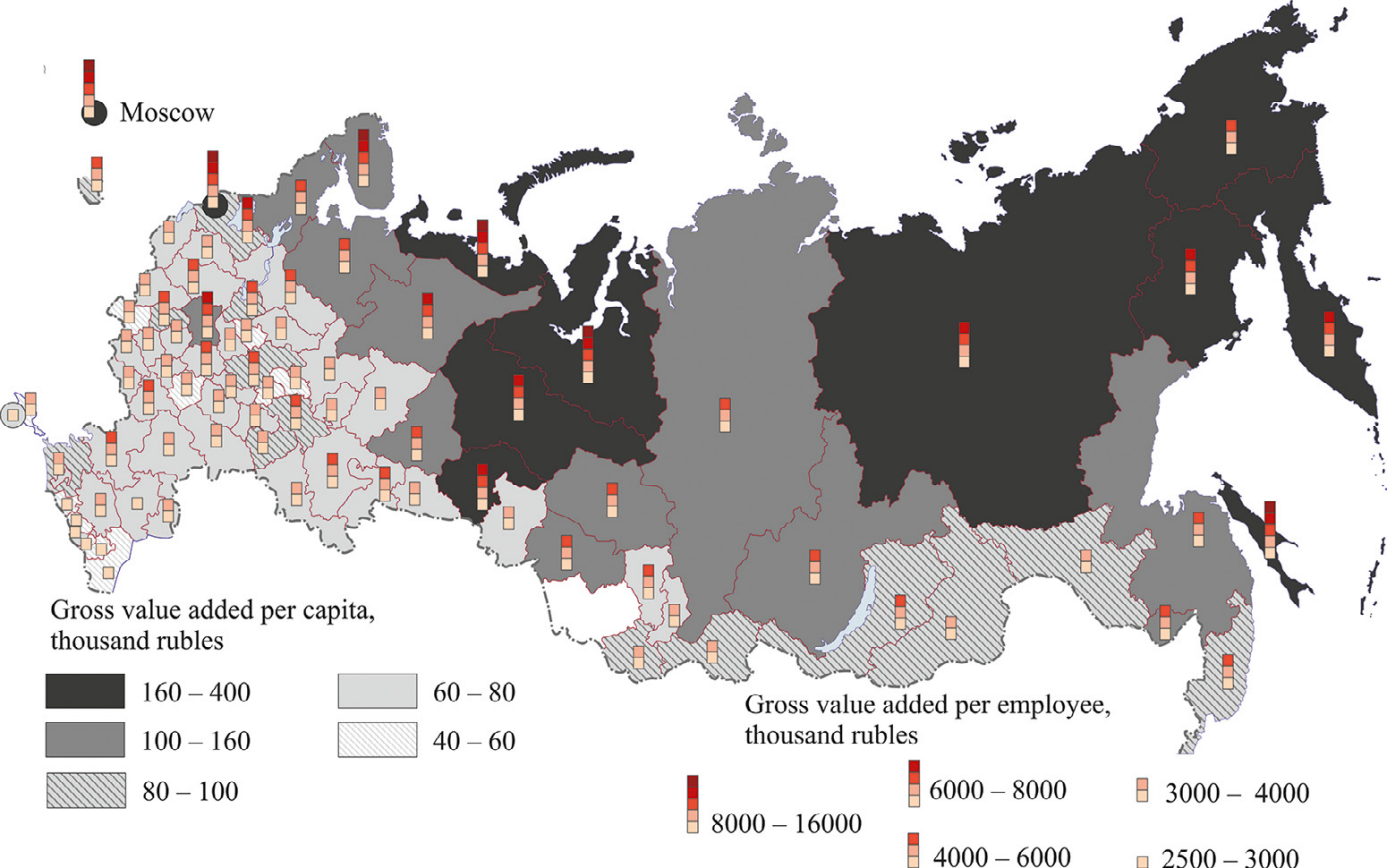
Gross value added per capita and per employee. Non-market services, 2018.

The grouping of regions according to the share of non-market services in GRP is similar to that of market services (Fig. 14, Table 9 Annex 3).). The leading group includes Moscow and Leningrad regions, the Tyumen region (without the autonomous regions), the Khanty-Mansi and Yamal-Nenets autonomous regions, the oil regions of Sakhalin and the Republic of Sakha (Yakutia). The latter also has a highly developed mining industry (diamonds, gold, etc.).

To summarize the analysis, we identified groups and subgroups of regions based on their typological characteristics, which are directly related to GRP figures, GRP sectoral composition and growth rates:

• GRP per capita;
• the share of the production of goods in GRP;
• gross value added per capita in the agricultural sector, in mining and manufacturing;
• GRP in 2018 as compared to 2004 (Table 3).

**Table 3:** Groups of Russian regions by GRP output, GRP sectoral composition and GRP per capita

			Value added per capita, thousand rubles, 2018			
Russian regions	GRP per capita, thousand rub., 2018	The share of the production of goods in GRP,%, 2018	Agriculture, forestry, hunting, fishing and fish farming	GRP per capita in 2018, as a percentage of 2004	Manufacturing	GRP per capita in 2018, as a percentage of 2004
*I. regions with a high share of services in GRP (postindustrial regions)*						

Advanced regions having highly developed services and the manufacturing industry 1.1. High GRP per capita and low GRP growth rates						
Moscow city	1423.6	22.9	1.9	0	239.1	121
1.2. High GRP per capita and high GRP growth rates						
St. Petersburg city	781.2	25.6	1.4	2.4	134.3	156
1.3. Average GRP per capita and high GRP growth rates						
Moscow region	556.4	31.2	9.7	0.9	114.5	155
*I. regions with a high share of the production of goods*						

2. Well-developed industrial-agrarian regions (agriculture and the manufacturing industries) having high GRP per capita, developed services and high growth rates						
Leningrad region	603.2	53.5	29.8	4.2	188.6	165
3. Mining regions having high GRP per capita						

3.1. Oil and gas regions having high GRP per capita						
Nenets Autonomous Area	6950.4	88.4	51.5	5780.6	13.4	…
Yamal-Nenets autonomous region	5710.1	82.7	6.1	3847.3	89.9	…
Khanty-Mansi autonomous region – Yugra	2680.1	83.4	5.7	1966.4	62.9	…
Sakhalin region	2407.9	80.4	74.4	1710.8	56.8	207
3.2. Mining regions having high GRP per capita						

3.2.A. Regions having accelerating GRP growth rates						
Republic of Sakha (Yakutia)	1123.1	68.6	17.6	578.8	12.6	151
3.2.B. Regions having developed fisheries and high GRP growth rates						
Magadan region	1196.7	58.3	74.9	455.3	14,3	165
Kamchatka Territory	750.4	51.2	163.9	42.3	109	170
3.2.С. Regions having low GRP growth rates						

Chukotka autonomous region	1578.5	64.5	40	636.1	4.5	130
4. Industrial mining and manufacturing regions having high GRP per capita						

4.1. High GRP growth rates						
Krasnoyarsk region	793	69.1	19.8	203.3	253.1	156
4.2.A. Decreasing GRP growth rates						
Komi Republic	796.8	65.7	11.6	351.5	91.5	127
Tyumen region	834.8	51.8	25.2	169.2	151.7	127*
4.2.B. Regions having developed fisheries and low GRP rates						
Murmansk region	642.7	49.6	92.5	77.1	73.9	120
5. Industrial and agrarian regions having average GRP per capita and high GRP growth rates						

5.1. Regions having agricultural, mining and manufacturing industries						
Belgorod region	559.2	63.6	102	108.2	104.3	232
Kursk region	385.6	60.2	70	44.6	64.8	186
Republic of Tatarstan	633.7	62.7	36	193.4	100.9	163
Orenburg region	507.8	72.8	36.4	208.5	71.5	152
5.2. Regions having mining and manufacturing industries, forestry and logging						
Irkutsk region	580.2	59.3	27.7	183.3	63.2	206
5.3. Regions having agricultural, mining, fish farming and fishing industries						
Astrakhan region	544.8	67.9	27.7	287.2	15.6	202
5.4.A regions having agricultural and manufacturing industries						
Lipetsk region	506.1	64.2	56.6	2.1	225.9	154
Kaluga region	461	55.9	26.5	1.6	195.2	201
Tula region	428.3	60.8	25.6	1.9	193	209
Republic of Bashkortostan	412.5	53.8	24.6	13.1	144.9	173
5.4.B. Regions having agricultural, manufacturing, forest and logging industries						
Novgorod region	434.2	55.6	33.7	1.9	144.3	175
6. Industrial regional having average GRP per capita						

6.1. Regions having mining and manufacturing industries and high GRP growth rates						
Perm region	503.8	62.9	10.9	112.7	153.9	150
6.2.A. Regions having mining and manufacturing industries and low GRP growth rates						
Republic of Karelia	451.4	51.2	27.7	77.3	93.6	127
Udmurt Republic	417.9	61.1	26.5	115.4	81.6	136
Samara region	473.8	55.4	18.5	98.7	104.9	122
Kemerovo region	462.5	64	8.9	169.9	78.9	121
Tomsk region	537.5	55.3	21	172.6	60.2	113
Republic of Khakassia	438.3	57.1	14.9	73.9	85.6	140
6.2. B. Regions having agricultural and manufacturing industries and low GRP growth rates						
Volgograd region	338.9	53.9	35.5	21.6	94	120
Omsk region	349.2	52.6	30.9	1.4	127.3	137
6.2.С. Regions having manufacturing industry and low GRP growth rates						
Vologda region	497	56.4	20.2	0.2	210	126
Chelyabinsk region	423	54	22.7	11.1	152.4	144
7. Agrarian regions with average GRP and high GRP growth rates						
Tambov region	323.6	52.8	80	0.1	42.3	215
8. Industrial-agrarian regions having manufacturing industry and GRP, which is lower than the Russian average, and high GRP growth rates						
Saratov region	290.6	50.7	30.4	11.3	60.7	173
Republic of Mordovia	284	52.5	39.8	0.1	74.2	196
Republic of Mari El	260.8	55.5	41.4	0.3	82.7	183
*III. Regions having a high share of services in GRP*						
9. Average GRP and high GRP growth rates						
Manufacturing regions						
Sverdlovsk region	527.2	46.2	12.8	8.5	171.7	170
Yaroslavl region	444	41.5	14.2	0.6	128.3	158.8
Nizhny Novgorod region	424.1	43.7	11.4	0.3	135.3	156.8
9.2. Regions having manufacturing, forestry and logging industries						
Arkhangelsk region	464.9	47	29.4	23.7	127.6	172,8*
9.3. Regions having agricultural, manufacturing, fish farming and fishing industries						
Kaliningrad region	461.6	45.7	29.2	15.3	104.5	171.7
9.4. Regions having agricultural and manufacturing industries						
Ryazan region	342.7	46.6	26.9	0.6	100.2	149
Voronezh region	404.8	40.4	55.8	1.9	59.8	195
Krasnodar region	416.8	33.6	44.1	4.9	48.8	159
Primorye region	437.1	27.5	36.7	5.5	41.3	153
Penza region	302.3	42.5	36.9	0.4	61.2	189
Rostov region	343.4	43.4	35.1	4.1	71.5	190
Orel region	310.4	46.7	61.6	0.4	47.9	154
9.5. Regions having the manufacturing industry						
Novosibirsk region	448.7	28.2	16.6	18	61.5	157
Smolensk region	330.8	44.5	15.5	0.9	71.9	171
Vladimir region	321.1	47.4	12.8	1.3	110.6	148
Tver region	345.9	44.6	23.5	0.4	76	155
9.6. Mining regions						
Trans-Baikal Territory	305.7	32.6	15.9	46	8.5	157
Jewish autonomous region	346.7	40	25.6	34.9	19.2	160
10. Regions having mining, manufacturing, forestry and logging industries, average GRP per capita and low GRP growth rates						

Khabarovsk region	536.4	32.3	34.2	37.2	53.2	135
Amur region	378.3	41.7	21.7	39.2	18.5	137
11. Regions having agricultural and manufacturing industries, low GRP per capita and high GRP growth rates						

Bryansk region	272.7	45.2	52.2	0.1	48.4	193
Stavropol Territory	255.7	41.9	37.6	1.4	35.6	156
Republic of Adygeya	238.8	41.1	32.6	3.9	40.1	216
Altay Territory	234.9	42.4	31.6	1.9	44.8	153
Republic of Daghestan	203.3	41.7	35.8	1.1	10.8	211
Kabardino-Balkarian Republic	168.2	45.5	32.3	0.5	18.2	167
Ulyanovsk region	280	46.3	18.1	3.6	75.7	157
Kurgan region	253.6	45.7	24	2	59.2	153
12. Regions having low GRP per capita and low GRP growth rates						

12.1. Regions having agricultural and manufacturing industries						
Kostroma region	281.6	45	21.1	0.3	66.9	142
Kirov region	260.3	46.1	20.9	0.8	76.4	140
Pskov region	259.4	41.2	31	1	44.1	143
Chuvash Republic	242.6	47.1	20.2	0.3	67.8	135
Ivanovo region	196	35.4	7.6	0.4	37.5	120
Republic of North Ossetia - Alania	185.6	30.1	22.2	0.6	13.4	136
Karachay-Cherkess Republic	165.4	48.6	32	3.6	19.3	141
Republic of Crimea	204.6	35.3	13.7	5.6	18.8	…
Republic of Buryatia	229.8	32.1	10.6	14.7	22.8	118
Sevastopol	180.1	26.3	6.1	3.4	15.7	…
12.2. Agricultural regions						
Republic of Kalmykia	268.9	35.4	69.6	3.4	2	128
Republic of Altay	231.5	30.9	27.7	2.8	10.6	127
12.3. Mining regions						
Republic of Tuva	212.9	39.2	11.4	53.1	1.6	121
13. Regions having the lowest GRP						

Chechen Republic	133.4	32	14.9	1.6	3.8	134⁎⁎
Republic of Ingushetia	112.6	36	14.3	1.9	6.1	152
Russia	578.7	46.4	25	85.4	104.7	148.6

Compiled by the authors based on the following data: Gross Regional Product per capita // EMISS https://fedstat.ru/indicator/42928;

Sectoral composition of Gross regional Product (OKVED 2) // EMISS https://www.fedstat.ru/indicator/59450.

⁎ the region and the autonomous region

⁎⁎ 2018 as a percentage by 2006.

The grouping was performed using the IBM SPSS Statistics 21 software. K-means clustering (with preliminary normalization of indicators) was used to form 13 groups within the 85 regions. Subdivision into subgroups was done by means of expert evaluation.

The proposed classification of regions can be used for comparative analysis of the geographical distribution of economic centers in Russia and the assessment of Russia's economy.

## 2 Experimental Design, Materials and Methods

Statistical data were obtained from the EMISS database of the Federal State Statistics Service of Russia and the *Regions of Russia* reference book. These data were used for computing average values of GRP per capita and per employee as well as chain indices of GRP growth rates. Coefficients of linear correlation between GRP and its growth rates, on the one hand, and other socio-economic characteristics of regions, on the other, were also calculated.

The data obtained are presented in three tables, three charts and 11 cartographic maps displaying quantitative indicators.

## Transparency document. Supporting information

Transparency data associated with this article can be found in the online version at http://dx.doi.org/10.17632/n36vrd8zrp.1

## Declaration of Competing Interest

The authors declare no competing interests.
